# Supplementary material for: Bispecific chimeric autoantibody receptor T cells eliminate acetylcholine receptor-specific B cells in myasthenia gravis models
Source: Nat Commun. 2026 Aug 20;17:8730. doi: 10.1038/s41467-026-76750-7 (PMC13493945; doi:10.1038/s41467-026-76750-7)
Supplement: Supplementary file 2 [file 41467_2026_76750_MOESM2_ESM.pdf]

nature portfolio

Peer Review File

Bispecific chimeric autoantibody receptor T cells for the elimination of acetylcholine receptor-specific B cells in myasthenia gravis

Corresponding Author: Dr Niels von Wardenburg

This file contains all reviewer reports in order by version, followed by all author rebuttals in order by version.

Version 0:

Reviewer comments:

Reviewer #1

(Remarks to the Author)
In their work Wardenberg et al. provide evidence of autoantibody specific CAR T cell, i.e. CAAR T directed against two AChR subunits efficiently depleting respective ab+ cells in vitro and in vivo.

The topic is timely and of interest for the filed of autoimmune disorders. The investigators use sophisticated tools, and answer almost all questions they set out to explore - up to the challenge of possible CAAR T cells saturation by soluble ab. The caveat remains in the principle therapeutic relevnce of CAAR T: how much of MG disease - and likewise other ab associated AID - is driven purely by the auto-ab? Following the reported success rate of CD19 CAR T in various AID at a considerable safety rate, the question on the long-term will be whether CAAR T can replace the alleged 'reset' after deep B cell depletion.

I recommend the publication of this work after revision: the authors should discuss the role of CAAR t in regard to aforementioned sweep of CD19, and lesser extend BCMA CAR T therapies in AID (given the economic advantage of 'off-the-shelf' tools, e.g. allogeneic CAR T, or in vivo approaches).

Reviewer #2

(Remarks to the Author)
Manuscript#: NCOMMS-26-020694

von Wardenburg et al conducted a strong study including a large number of comprehensive laboratory experiments in vitro and in vivo (animal). The rationale was based on robust background knowledge from both past research studies on MG and new technology in rapid development.

Summary – I suggest that the authors make clear that “in vivo” is not in humans but is experimental.

Introduction – Clear and focused; I have no specific or relevant comments.

Results and Discussion – some points require clarification and improvement:
1. Lines 110-111: please explain (probably in the methods session) what is “full-length AChR expressed in HEK cells.” - is it all the 5 subunits including intra and extracellular domains? and also just add if with or without rapsyn.
2. Lines 195-197: “Together, these findings demonstrate …, although they can mediate minimal cytotoxicity toward Fc receptor expressing cells in an artificial system. Relevance of this finding will be monitored in future clinical trials” - I advise cautious re-writing this last sentence as one cannot indicate or expect that the relevance of laboratory findings will be monitored in clinical trials – there must be other studies in the meantime.
3. Lines 240-241: “These findings demonstrate that both mixed and co-transduced AChR CAAR T cells effectively eliminate

AChRα1- and AChRβ1-reactive B cells in vivo while maintaining overall safety within the model constraints.” - Although this statement about safety is related to an in vivo model, thus acceptable in the context, I suggest adding a note of uncertainty regarding safety in humans, based on the current study.
4. Lines 264-266: “Patient sera that bound to the AChRa1 CAAR predominantly came from individuals with symptomatic MG and high autoantibody titers suggesting that these patients may derive the greatest benefit from AChR CAAR T therapy.” I recommend that this statement is changed; although the results only seem to suggest that a subset of patients may derive the greatest benefit from AChR CAAR T therapy, authors should emphasise that this research study did not aim to identify patients who could potentially benefit from this therapy.
5. Similarly, some paragraphs below (lines 290-291), where it says “…. will be crucial for the successful translation of this technology into clinical application” should be cautiously phrased.

Methods – very good and well described, detailed and quoted.

Figures and Tables and respective legends – very good.

References – appropriate

In summary, the methods and findings here described and discussed are robust, and although the study has limitations, acknowledged and briefly discussed by the authors, overall, it adds great value to the field of future management strategies in autoantibody mediated autoimmunity in humans. The authors suggest that some of the study findings and their interpretation are translatable to the human disease. However, more caution is advised when writing about very encouraging results from laboratory and limited experimental studies and their potential application in human disease.

Version 1:

Reviewer comments:

Reviewer #1

(Remarks to the Author)
The authors have addressed my comments sufficiently. No further comments.

Open Access This Peer Review File is licensed under a Creative Commons Attribution 4.0 International License, which permits use, sharing, adaptation, distribution and reproduction in any medium or format, as long as you give appropriate credit to the original author(s) and the source, provide a link to the Creative Commons license, and indicate if changes were made.
In cases where reviewers are anonymous, credit should be given to 'Anonymous Referee' and the source.
The images or other third party material in this Peer Review File are included in the article’s Creative Commons license, unless indicated otherwise in a credit line to the material. If material is not included in the article’s Creative Commons license and your intended use is not permitted by statutory regulation or exceeds the permitted use, you will need to obtain

permission directly from the copyright holder.
To view a copy of this license, visit https://creativecommons.org/licenses/by/4.0/

Point-by-point response to the reviewers' comments

Reviewer #1:

In their work Wardenburg et al. provide evidence of autoantibody specific CAR T cell, i.e. CAAR T directed against two AChR subunits efficiently depleting respective ab+ cells in vitro and in vivo.

The topic is timely and of interest for the field of autoimmune disorders. The investigators use sophisticated tools, and answer almost all questions they set out to explore - up to the challenge of possible CAAR T cells saturation by soluble ab.

Response: We thank the reviewer for the careful reading of the manuscript and the positive overall statement.

The caveat remains in the principle therapeutic relevance of CAAR T: how much of MG disease - and likewise other ab associated AID - is driven purely by the auto-ab?

Response:

We revised the discussion to address the relevance of non-B cell populations in MG pathology and consequences for the therapeutic relevance of the CAAR T cell approach, which now reads (please see page 13, line 316-321):

Whether non-B cell immune populations substantially contribute to disease pathology also remains uncertain. Notably, recent studies of CD19- and BCMA-directed CAR T cell therapies have demonstrated remarkable efficacy in MG [1-3], and immune profiling following BCMA CAR T cell treatment revealed reduced inflammatory signatures across multiple immune cell compartments [2], suggesting that elimination of pathogenic B cells may be sufficient to restore broader immune homeostasis.

Following the reported success rate of CD19 CAR T in various AID at a considerable safety rate, the question on the long-term will be whether CAAR T can replace the alleged 'reset' after deep B cell depletion.

Response:

We agree with the reviewer that an important unresolved question is whether antigen-specific CAAR T cell therapy can ultimately match the durability and breadth of remission achieved by the more profound immune reset associated with CD19- or BCMA-directed CAR T cell therapies. Clinical studies will be required to determine whether selective depletion of autoreactive B cells is sufficient to induce sustained disease control across the heterogeneous spectrum of MG. The safety profile of CD19- or BCMA-CAR T cell therapy in AID is surprisingly favorable despite the deep depletion of B cells and ASCs which is frequently accompanied by hypogammaglobulinemia. However, infections are common complications of CAR T cell therapy in oncologic patients and have also been reported in MG patients [1, 4]. In this context, CAAR T cell therapy may offer an approach that preserves the therapeutic benefits of targeted B cell depletion while potentially reducing the risks associated with broad and prolonged immunosuppression.

Therefore, we added the following sentences to the discussion (see page 13, lines 325-329):

Whether AChR CAAR T cells can achieve pathogenic B cell depletion of comparable depth and durability remains an important question. Ongoing and future clinical studies will determine which platform offers the optimal balance of efficacy, safety, practicality, and cost for AChR MG.

I recommend the publication of this work after revision: the authors should discuss the role of CAAR t in regard to aforementioned sweep of CD19, and lesser extend BCMA CAR T therapies in AID (given the economic advantage of 'off-the-shelf' tools, e.g. allogeneic CAR T, or in vivo approaches).

Response: We thank the reviewer for their positive assessment of our work and support for publication of this manuscript. The additional paragraph addressing competing technologies like CD19 or BCMA CAR T cells, in vivo and allogenic CAR T cells as well as T cell engagers in the revised manuscript now reads (please see page 13, lines 303-310):

Collectively, these findings support AChR CAAR T cells as a promising precision immunotherapy for patients with MG and support their advancement into first-in-human clinical evaluation. Their ultimate clinical role will need to be considered alongside other emerging cell-based approaches, including CD19 and BCMA CAR T cells, allogeneic and in vivo CAR T platforms [39, 40], and bispecific T cell engagers [41]. Whether AChR CAAR T cells can achieve pathogenic B cell depletion of comparable depth and durability remains an important question. Ongoing and future clinical studies will determine which platform offers the optimal balance of efficacy, safety, practicality, and cost for AChR MG.

Reviewer #2:

von Wardenburg et al conducted a strong study including a large number of comprehensive laboratory experiments in vitro and in vivo (animal). The rationale was based on robust background knowledge from both past research studies on MG and new technology in rapid development.

Response: We thank the reviewer for the careful reading of the manuscript and the positive overall statement.

Summary – I suggest that the authors make clear that "in vivo" is not in humans but is experimental.

Response:

To avoid misunderstanding the phrase now reads (please see page 2, line 36):

In a xenograft mouse model, they depleted pathogenic B cell lines and reduced autoantibody levels in the circulation and at the neuromuscular junction.

Introduction – Clear and focused; I have no specific or relevant comments.

Results and Discussion – some points require clarification and improvement:

1. Lines 110-111: please explain (probably in the methods session) what is "full-length AChR expressed in HEK cells." - is it all the 5 subunits including intra and extracellular domains? and also just add if with or without rapsyn.

Response: We added to the method section "Binding of recombinant autoantibodies and sera by flow cytometry" the following sentence (please see page 43, lines 732-733): "...using HEK293 cells transiently transfected with plasmids encoding for AChRα1, AChRβ1, AChRδ, AChRε and rapsyn-eGFP.

2. Lines 195-197: “Together, these findings demonstrate …, although they can mediate minimal cytotoxicity toward Fc receptor expressing cells in an artificial system. Relevance of this finding will be monitored in future clinical trials” - I advise cautious re-writing this last sentence as one cannot indicate or expect that the relevance of laboratory findings will be monitored in clinical trials – there must be other studies in the meantime.

Response: We carefully rephrased the sentence as follows (please see page 9, lines 202-203): The relevance of this finding warrants further preclinical investigation, for example through assessment in an in vitro model using patient-derived PBMCs.

3. Lines 240-241: “These findings demonstrate that both mixed and co-transduced AChR CAAR T cells effectively eliminate AChRα1- and AChRβ1-reactive B cells in vivo while maintaining overall safety within the model constraints.” - Although this statement about safety is related to an in vivo model, thus acceptable in the context, I suggest adding a note of uncertainty regarding safety in humans, based on the current study.

Response: To address the limited translatability of the safety findings we added the following sentence at the end of the paragraph (please see page 11, lines 250-251): Nevertheless, additional safety evaluations, including assessment of potential off-target toxicity, are warranted prior to clinical application in humans.

4. Lines 264-266: “Patient sera that bound to the AChRa1 CAAR predominantly came from individuals with symptomatic MG and high autoantibody titers suggesting that these patients may derive the greatest benefit from AChR CAAR T therapy.” I recommend that this statement is changed; although the results only seem to suggest that a subset of patients may derive the greatest benefit from AChR CAAR T therapy, authors should emphasize that this research study did not aim to identify patients who could potentially benefit from this therapy.

Response: As suggested by the reviewer, we added a sidenote, that in our study we did not systematically address the question, which patient would benefit the most from CAAR therapy. It now reads as (changes in orange) (please see page 12, lines 276):

Patient sera that bound to the AChRa1 CAAR predominantly came from individuals with symptomatic MG and high autoantibody titers suggesting that these patients may derive the greatest benefit from AChR CAAR T therapy, although this was not systematically examined.

5. Similarly, some paragraphs below (lines 290-291), where it says “…. will be crucial for the successful translation of this technology into clinical application” should be cautiously phrased.

Response: We cautiously rephrased the sentence (changes in orange) (please see page 13, line 301-302):

The efficient engraftment, persistence, and stable CAAR expression demonstrated here are positive indicators for the successful translation of this technology into clinical application.

Methods – very good and well described, detailed and quoted.

Figures and Tables and respective legends – very good.

References – appropriate

In summary, the methods and findings here described and discussed are robust, and although the study has limitations, acknowledged and briefly discussed by the authors, overall, it adds great value to the field of future management strategies in autoantibody mediated autoimmunity in humans. The authors suggest that some of the study findings and their interpretation are translatable to the human disease. However, more caution is advised when writing about very encouraging results from laboratory and limited experimental studies and their potential application in human disease.

Response: We thank the reviewer for the constructive, overall positive, feedback and for highlighting the need for greater caution when extrapolating from laboratory or experimental findings. The manuscript has been revised accordingly (see beforementioned responses).

1. Vu, T., et al., *BCMA-directed mRNA CAR T cell therapy for myasthenia gravis: a randomized, double-blind, placebo-controlled phase 2b trial.* Nature Medicine, 2026. **32**(3): p. 1131-1141.
2. Tian, D.S., et al., *B cell lineage reconstitution underlies CAR-T cell therapeutic efficacy in patients with refractory myasthenia gravis.* EMBO Mol Med, 2024. **16**(4): p. 966-987.
3. Haghikia, A., et al., *Anti-CD19 CAR T cells for refractory myasthenia gravis.* Lancet Neurol, 2023. **22**(12): p. 1104-1105.
4. Dye, B., et al., *Infectious complications of chimeric antigen receptor T cell therapy and bispecific antibodies - A review.* Am J Med Sci, 2025.
