# Supplementary material for: Bispecific chimeric autoantibody receptor T cells eliminate acetylcholine receptor-specific B cells in myasthenia gravis models
Source: Nat Commun. 2026 Aug 20;17:8730. doi: 10.1038/s41467-026-76750-7 (PMC13493945; doi:10.1038/s41467-026-76750-7)
Supplement: Supplementary file 3 [file 41467_2026_76750_MOESM3_ESM.pdf]

nature portfolio

Corresponding author(s): Niels von Wardenburg, Harald Prüss

Last updated by author(s): Jul 22, 2026

Reporting Summary

Nature Portfolio wishes to improve the reproducibility of the work that we publish. This form provides structure for consistency and transparency in reporting. For further information on Nature Portfolio policies, see our Editorial Policies and the Editorial Policy Checklist.

Statistics

For all statistical analyses, confirm that the following items are present in the figure legend, table legend, main text, or Methods section.

n/a	Confirmed	
☐	☒	The exact sample size (*n*) for each experimental group/condition, given as a discrete number and unit of measurement
☐	☒	A statement on whether measurements were taken from distinct samples or whether the same sample was measured repeatedly
☐	☒	The statistical test(s) used AND whether they are one- or two-sided<br>*Only common tests should be described solely by name; describe more complex techniques in the Methods section.*
☐	☒	A description of all covariates tested
☐	☒	A description of any assumptions or corrections, such as tests of normality and adjustment for multiple comparisons
☐	☒	A full description of the statistical parameters including central tendency (e.g. means) or other basic estimates (e.g. regression coefficient) AND variation (e.g. standard deviation) or associated estimates of uncertainty (e.g. confidence intervals)
☐	☒	For null hypothesis testing, the test statistic (e.g. *F*, *t*, *r*) with confidence intervals, effect sizes, degrees of freedom and *P* value noted<br>*Give P values as exact values whenever suitable.*
☒	☐	For Bayesian analysis, information on the choice of priors and Markov chain Monte Carlo settings
☒	☐	For hierarchical and complex designs, identification of the appropriate level for tests and full reporting of outcomes
☐	☒	Estimates of effect sizes (e.g. Cohen's *d*, Pearson's *r*), indicating how they were calculated

Our web collection on statistics for biologists contains articles on many of the points above.

Software and code

Policy information about availability of computer code

Data collection	BD FACSDiva BD Biosciences<br>Spectramax ID3 Molecular Devices<br>SoftMax Pro version 7.1 Molecular Devices<br>Nikon elements 5.2 software<br>FlowLogic software Inivai Technologies
Data analysis	Flowjo version 10.7.2 BD Biosciences<br>GraphPad Prism, version 9.5.1 GraphPad Software<br>Microsoft Excel, Word, PowerPoint Microsoft Office<br>Snapgene version 5.0.8 Dotmatics<br>NIH ImageJ 1.52i

For manuscripts utilizing custom algorithms or software that are central to the research but not yet described in published literature, software must be made available to editors and reviewers. We strongly encourage code deposition in a community repository (e.g. GitHub). See the Nature Portfolio guidelines for submitting code & software for further information.

Data

Policy information about availability of data

All manuscripts must include a data availability statement. This statement should provide the following information, where applicable:

- Accession codes, unique identifiers, or web links for publicly available datasets
- A description of any restrictions on data availability
- For clinical datasets or third party data, please ensure that the statement adheres to our policy

The authors declare that the data supporting the findings of this study are available within the paper and its supplementary information files. The main raw data generated in this study (Fig. 1-5) have been deposited in Figshare.com database under accession code ACHRCAART-2026 [add hyperlink here]. All data included in the figure graphs (Fig. 1-5, Supplementary Figure 1-6) of this study are provided in the Source Data file.

Research involving human participants, their data, or biological material

Policy information about studies with human participants or human data. See also policy information about sex, gender (identity/presentation), and sexual orientation and race, ethnicity and racism.

Reporting on sex and gender	For the assessment of MG serum binding to CAAR T cells, 53% of the samples were collected from female patients. No sex-specific analyses were performed because the sample size was insufficient to reliably detect potential sex-related differences in binding.<br><br>Four AChR antibody sequences were derived from male patients and six from female patients.<br><br>Animal studies were conducted exclusively in female NOG mice, as male NOG mice are known to exhibit aggressive behavior and are difficult to group-house safely and consistently.
Reporting on race, ethnicity, or other socially relevant groupings	As serum samples from MG patients were used exclusively to assess binding to AChRa1- or AChRb1-CAAR-expressing cells, reporting race, ethnicity, or other socially relevant demographic groupings was not considered relevant to the objectives of this analysis.
Population characteristics	See above
Recruitment	Samples from the Berlin cohort were selected based on high AChR antibody titers, whereas samples from the Florence cohort were selected to represent a range of low, intermediate, and high AChR antibody titers.
Ethics oversight	All primary samples (healthy donor and patient material) were obtained after informed consent and analyses were approved by the Institutional Review Board of Charité - Universitätsmedizin Berlin (study protocol number EA1/258/18), and the University of Florence/Careggi University Hospital (Florence, Italy) with protocol n. 22914_bio.

Note that full information on the approval of the study protocol must also be provided in the manuscript.

Field-specific reporting

Please select the one below that is the best fit for your research. If you are not sure, read the appropriate sections before making your selection.

☒ Life sciences ☐ Behavioural & social sciences ☐ Ecological, evolutionary & environmental sciences

For a reference copy of the document with all sections, see nature.com/documents/nr-reporting-summary-flat.pdf

Life sciences study design

All studies must disclose on these points even when the disclosure is negative.

Sample size	No formal sample size calculation was performed. For the animal studies, the selected sample size was considered sufficient because a large effect size for CAAR T cell-mediated in vivo killing was anticipated based on findings from previous related studies.
Data exclusions	No data were excluded from the analysis
Replication	Key experiments were performed at least in three independent replicates to ensure reproducability. Animal studies were not replicated for ethical reasons.
Randomization	Animals were randomly assigned to treatment groups.
Blinding	Blinding was applied during the quantitative analysis of histological images. For assays analyzed according to predefined criteria, blinding was not performed.

Reporting for specific materials, systems and methods

We require information from authors about some types of materials, experimental systems and methods used in many studies. Here, indicate whether each material, system or method listed is relevant to your study. If you are not sure if a list item applies to your research, read the appropriate section before selecting a response.

Materials & experimental systems

n/a	Involved in the study
☐	☒ Antibodies
☐	☒ Eukaryotic cell lines
☐	☐ Palaeontology and archaeology
☐	☒ Animals and other organisms
☐	☒ Clinical data
☐	☐ Dual use research of concern
☐	☐ Plants

Methods

n/a	Involved in the study
☐	☐ ChIP-seq
☐	☒ Flow cytometry
☐	☐ MRI-based neuroimaging

Antibodies

Antibodies used

mouse CD45-PE-Vio® 615 Miltenyi Biotec REA737 130-110-666, 1:100, Clone REA73
human CD45-VioBlue® Miltenyi Biotec REA747 130-110-637, 1:100, Clone REA747
human CD3-VioGreen™ Miltenyi Biotec REA613 130-113-142, 1:100, Clone REA613
human CD4-Vio® Bright V600 Miltenyi Biotec REA623 130-129-579, 1:100, Clone REA 623
human CD8-APC-Vio® 770 Miltenyi Biotec REA734 130-110-681, 1:100, Clone REA734
donkey anti-rabbit IgG Jackson ImmunoResearch Cat#711-005-152; RRID:AB_2340585, 10 ug/mL
donkey anti-human IgG-HRPO Jackson ImmunoResearch Cat#709-035-149; RRID:AB_2340495; 1:5000
anti-human IgG AF647 Thermo Fisher Scientific Cat#A21445; RRID:AB_2535862, 1:400
anti-EGFR antibody cetuximab-PE R&D Systems clone:Hu1 Cat#FAB9577P: RRID:AB_2942015, 1:10
goat anti-human IgG Fc specific unconjugated antibody Invitrogen Cat#31125; RRID:AB_429669; 1:200
donkey anti-goat IgG (H+L) AF647-conjugated antibody Invitrogen Cat#A-21447; RRID:AB_2535864; 1:800
biotinylated cetuximab R&D Systems Hu1 Cat#FAB9577B; RRID:AB_2942016; 1:10
CD3-PerCP Biolegend UCHT1 Cat#300428; RRID:AB_893298; 1:20
CD69-BV510 Biolegend REA734 Cat#310936; RRID:AB_2563834; 1:20
anti-rat IgG Alexa Fluor 594-conjugated antibody Cat# A11007; 1:500
cetuximab-AF647 R&D Systems Hu1 Cat#FAB9577R; 1:10
mAb35 Hölzel Diagnostika Cat#HY-P990302, 10 µg/mL
anti-human IgG Alexa Fluor 488-conjugated antibody Dianova Cat#109-545-003; 1:500

Validation

Validation of individual antibodies was stated on manufacturers homepages.

Eukaryotic cell lines

Policy information about cell lines and Sex and Gender in Research

Cell line source(s)

K562 cells were kindly provided by Maja Milanovic (Charité Universitätsmedizin Berlin)
Jurkat cells were obtained from Cytion, cat#302147
Nalm6 cells were obtained from Cytion, cat.#300297
HEK293T cells were obtained from DSMZ (acc. no. ACC 305)
THP-1 were kindly provided by Daniel Berchtold (Charité Universitätsmedizin Berlin)
G10 hybridoma cells were kindly provided by the German Rheumatology Research Center (DRFZ)

Authentication

K562 and THP-1 cells were not authenticated. Jurkat, Nalm6 and HEK293T cells were authenticated by the respective provider.

Mycoplasma contamination

Mycoplasma contamination was routinely monitored and ruled out.

Commonly misidentified lines
(See ICLAC register)

n.a.

Palaeontology and Archaeology

Specimen provenance	*Provide provenance information for specimens and describe permits that were obtained for the work (including the name of the issuing authority, the date of issue, and any identifying information). Permits should encompass collection and, where applicable, export.*
Specimen deposition	*Indicate where the specimens have been deposited to permit free access by other researchers.*
Dating methods	*If new dates are provided, describe how they were obtained (e.g. collection, storage, sample pretreatment and measurement), where they were obtained (i.e. lab name), the calibration program and the protocol for quality assurance OR state that no new dates are provided.*

☐ Tick this box to confirm that the raw and calibrated dates are available in the paper or in Supplementary Information.

Ethics oversight	*Identify the organization(s) that approved or provided guidance on the study protocol, OR state that no ethical approval or guidance was required and explain why not.*

Note that full information on the approval of the study protocol must also be provided in the manuscript.

Animals and other research organisms

Policy information about studies involving animals; ARRIVE guidelines recommended for reporting animal research, and Sex and Gender in Research

Laboratory animals	Female NOD.Cg-Prkdcscid Il2rgtm1Sug/JicTac mice (NOG, 6–8 weeks; Taconic). Animals were maintained on a 12-hour light/dark cycle at a constant temperature of 23°C, with food and water available ad libitum.
Wild animals	*Provide details on animals observed in or captured in the field; report species and age where possible. Describe how animals were caught and transported and what happened to captive animals after the study (if killed, explain why and describe method; if released, say where and when) OR state that the study did not involve wild animals.*
Reporting on sex	Animal studies were conducted exclusively in female NOG mice, as male NOG mice are known to exhibit aggressive behavior and are difficult to group-house safely and consistently.
Field-collected samples	*For laboratory work with field-collected samples, describe all relevant parameters such as housing, maintenance, temperature, photoperiod and end-of-experiment protocol OR state that the study did not involve samples collected from the field.*
Ethics oversight	Federal Office for Health and Social Affairs, Berlin, Germany; approval number: vereinf. Genehm. E0023/23

Note that full information on the approval of the study protocol must also be provided in the manuscript.

Clinical data

Policy information about clinical studies

All manuscripts should comply with the ICMJE guidelines for publication of clinical research and a completed CONSORT checklist must be included with all submissions.

Clinical trial registration	*Provide the trial registration number from ClinicalTrials.gov or an equivalent agency.*
Study protocol	*Note where the full trial protocol can be accessed OR if not available, explain why.*
Data collection	*Describe the settings and locales of data collection, noting the time periods of recruitment and data collection.*
Outcomes	*Describe how you pre-defined primary and secondary outcome measures and how you assessed these measures.*

Dual use research of concern

Policy information about dual use research of concern

Hazards

Could the accidental, deliberate or reckless misuse of agents or technologies generated in the work, or the application of information presented in the manuscript, pose a threat to:

No | Yes
- [ ] [ ] Public health
- [ ] [ ] National security
- [ ] [ ] Crops and/or livestock
- [ ] [ ] Ecosystems
- [ ] [ ] Any other significant area

Experiments of concern

Does the work involve any of these experiments of concern:

No | Yes
- [ ] [ ] Demonstrate how to render a vaccine ineffective
- [ ] [ ] Confer resistance to therapeutically useful antibiotics or antiviral agents
- [ ] [ ] Enhance the virulence of a pathogen or render a nonpathogen virulent
- [ ] [ ] Increase transmissibility of a pathogen
- [ ] [ ] Alter the host range of a pathogen
- [ ] [ ] Enable evasion of diagnostic/detection modalities
- [ ] [ ] Enable the weaponization of a biological agent or toxin
- [ ] [ ] Any other potentially harmful combination of experiments and agents

Plants

Seed stocks	*Report on the source of all seed stocks or other plant material used. If applicable, state the seed stock centre and catalogue number. If plant specimens were collected from the field, describe the collection location, date and sampling procedures.*
Novel plant genotypes	*Describe the methods by which all novel plant genotypes were produced. This includes those generated by transgenic approaches, gene editing, chemical/radiation-based mutagenesis and hybridization. For transgenic lines, describe the transformation method, the number of independent lines analyzed and the generation upon which experiments were performed. For gene-edited lines, describe the editor used, the endogenous sequence targeted for editing, the targeting guide RNA sequence (if applicable) and how the editor was applied.*
Authentication	*Describe any authentication procedures for each seed stock used or novel genotype generated. Describe any experiments used to assess the effect of a mutation and, where applicable, how potential secondary effects (e.g. second site T-DNA insertions, mosiacism, off-target gene editing) were examined.*

ChIP-seq

Data deposition

- [ ] Confirm that both raw and final processed data have been deposited in a public database such as GEO.
- [ ] Confirm that you have deposited or provided access to graph files (e.g. BED files) for the called peaks.

Data access links<br>*May remain private before publication.*	*For "Initial submission" or "Revised version" documents, provide reviewer access links. For your "Final submission" document, provide a link to the deposited data.*
Files in database submission	*Provide a list of all files available in the database submission.*
Genome browser session<br>(e.g. UCSC)	*Provide a link to an anonymized genome browser session for "Initial submission" and "Revised version" documents only, to enable peer review. Write "no longer applicable" for "Final submission" documents.*

Methodology

Replicates	*Describe the experimental replicates, specifying number, type and replicate agreement.*
Sequencing depth	*Describe the sequencing depth for each experiment, providing the total number of reads, uniquely mapped reads, length of reads and whether they were paired- or single-end.*
Antibodies	*Describe the antibodies used for the ChIP-seq experiments; as applicable, provide supplier name, catalog number, clone name, and lot number.*
Peak calling parameters	*Specify the command line program and parameters used for read mapping and peak calling, including the ChIP, control and index files used.*
Data quality	*Describe the methods used to ensure data quality in full detail, including how many peaks are at FDR 5% and above 5-fold enrichment.*

Software | *Describe the software used to collect and analyze the ChIP-seq data. For custom code that has been deposited into a community repository, provide accession details.*

Flow Cytometry

Plots

Confirm that:

- [x] The axis labels state the marker and fluorochrome used (e.g. CD4-FITC).
- [x] The axis scales are clearly visible. Include numbers along axes only for bottom left plot of group (a 'group' is an analysis of identical markers).
- [x] All plots are contour plots with outliers or pseudocolor plots.
- [x] A numerical value for number of cells or percentage (with statistics) is provided.

Methodology

Sample preparation	Blood samples were collected via retrobulbar puncture into EDTA tubes (flow cytometry) or lithium-heparin tubes (serum). Serum was stored at −80 °C, while EDTA blood samples were analyzed immediately. Single-cell suspensions from spleens were generated by mechanical dissociation through a 70 µm strainer and resuspended in PBS containing 2 mM EDTA and 0.5% BSA.
Instrument	FACSCanto II (BD Biosciences, NJ, USA), MACSQuant Analyzer (Miltenyi Biotec, Germany), MACSQuant16 (Miltenyi Biotec)
Software	FlowJo v10.9.0
Cell population abundance	n.a.
Gating strategy	FSC/SSC gates were set broadly to avoid excluding data points based on morphological variation. Gates were defined using negative controls.

- [x] Tick this box to confirm that a figure exemplifying the gating strategy is provided in the Supplementary Information.

Magnetic resonance imaging

Experimental design

Design type	*Indicate task or resting state; event-related or block design.*
Design specifications	*Specify the number of blocks, trials or experimental units per session and/or subject, and specify the length of each trial or block (if trials are blocked) and interval between trials.*
Behavioral performance measures	*State number and/or type of variables recorded (e.g. correct button press, response time) and what statistics were used to establish that the subjects were performing the task as expected (e.g. mean, range, and/or standard deviation across subjects).*

Acquisition

Imaging type(s)	*Specify: functional, structural, diffusion, perfusion.*
Field strength	*Specify in Tesla*
Sequence & imaging parameters	*Specify the pulse sequence type (gradient echo, spin echo, etc.), imaging type (EPI, spiral, etc.), field of view, matrix size, slice thickness, orientation and TE/TR/flip angle.*
Area of acquisition	*State whether a whole brain scan was used OR define the area of acquisition, describing how the region was determined.*

Diffusion MRI ☐ Used ☐ Not used

Preprocessing

Preprocessing software	*Provide detail on software version and revision number and on specific parameters (model/functions, brain extraction, segmentation, smoothing kernel size, etc.).*
Normalization	*If data were normalized/standardized, describe the approach(es): specify linear or non-linear and define image types used for transformation OR indicate that data were not normalized and explain rationale for lack of normalization.*
Normalization template	*Describe the template used for normalization/transformation, specifying subject space or group standardized space (e.g. original Talairach, MNI305, ICBM152) OR indicate that the data were not normalized.*

Noise and artifact removal	*Describe your procedure(s) for artifact and structured noise removal, specifying motion parameters, tissue signals and physiological signals (heart rate, respiration).*
Volume censoring	*Define your software and/or method and criteria for volume censoring, and state the extent of such censoring.*

Statistical modeling & inference

Model type and settings	*Specify type (mass univariate, multivariate, RSA, predictive, etc.) and describe essential details of the model at the first and second levels (e.g. fixed, random or mixed effects; drift or auto-correlation).*
Effect(s) tested	*Define precise effect in terms of the task or stimulus conditions instead of psychological concepts and indicate whether ANOVA or factorial designs were used.*

Specify type of analysis: ☐ Whole brain ☐ ROI-based ☐ Both

Statistic type for inference<br>(See Eklund et al. 2016)	*Specify voxel-wise or cluster-wise and report all relevant parameters for cluster-wise methods.*
Correction	*Describe the type of correction and how it is obtained for multiple comparisons (e.g. FWE, FDR, permutation or Monte Carlo).*

Models & analysis

n/a	Involved in the study
☐	☐ Functional and/or effective connectivity
☐	☐ Graph analysis
☐	☐ Multivariate modeling or predictive analysis

Functional and/or effective connectivity	*Report the measures of dependence used and the model details (e.g. Pearson correlation, partial correlation, mutual information).*
Graph analysis	*Report the dependent variable and connectivity measure, specifying weighted graph or binarized graph, subject- or group-level, and the global and/or node summaries used (e.g. clustering coefficient, efficiency, etc.).*
Multivariate modeling and predictive analysis	*Specify independent variables, features extraction and dimension reduction, model, training and evaluation metrics.*
